# Bromide of Potassium

**Published:** 1875-01

**Authors:** W. F. Barr

**Affiliations:** Abingdon, Virginia


### BROMIDE OF POTASSIUM.

BY W. F. BARR, M.D., OF ABINGDON, VIRGINIA.

(*Read before the Abingdon Academy of Medicine. Published by Request.*)

Although this medicine was first described by Ballard in 1826, and has been prescribed by a few physicians since that time, yet it is within the past few years that its therapeutical effects have been in the least understood. In an edition of Chapman's Therapeutics before me, this medicine is not mentioned; and although the London Pharmacopœia of 1836 gives a formula for its preparation, yet it is omitted in that of 1851!

In the United States Dispensatory of 1843, by Wood & Bache, it is stated that "bromide of potassium is deemed alterative and resolvent." It is only alluded to by the late learned Dr. Dunglison, of Philadelphia, in his excellent work on Materia Medica and Therapeutics of 1850; and in his work on New Remedies, edition of 1851, he gives but little information about it. In each of his works he merely states that its "therapeutical effects were found to be identical with iodide of potassium." Pereira, in his full and elaborate work on Materia Medica and Therapeutics (edition of 1852), says: "The effects of bromide of potassium appear to be analagous to those of iodide of potassium."

Bromine appears in the secondary list of the Pharmacopœia of the United States of 1842.

Bromide of potassium seems to have been first used by Pouché in 1828. He employed it with benefit in the treatment of bronchocele, goitre, scrofulous tumors, chronic otorrhœa, and scrofulous tumefaction of the testicle. From Dunglison's Materia Medica and Therapeutics we learn that Magendie employed this medicine "in scrofula, in amenorrhœa, and in hypertrophy of the ventricles of the heart. Prieger recommended an ointment in inveterate *porrigo favosa*, as well as in obstinate and malignant tetter, with good success. Dr. Williams used it with good success in the case of diseases of the spleen." Ricord was, at one time, on account of "the *low* price of the bromide compared with that of the iodide," induced to substitute it for iodide of potassium in the treatment of secondary syphilitic affections.

But little more was known or done with this medicine until it was brought forward and introduced into practice by Dr. C. E. Brown-Sequard, at this time an Honorary Fellow of our Association. Dr. James Turnbull, of the Liverpool Royal Infirmary, states that "Brown-Sequard was the first to point out the value of bromide of potassium in inducing sleep, and to show the mode of giving it, and the kind of cases in which it is most likely to prove of service."

Dr. Brown-Sequard, in one of his lectures on the diagnosis and treatment of functional nervous affections, says: "It is of the utmost importance to improve the sleep, which is generally bad in persons attacked with a morbid increase of the reflex excitability. For this purpose an invaluable remedy has recently been discovered; it is the bromide of potassium. Except when pain is one of the causes preventing sleep (in which case opium, aconite and belladonna should be employed together), I have found that this remedy has a wonderful power to produce quiet and refreshing sleep, without any drawback that I am aware of. I usually give to adults a dose of thirty grains of that salt a quarter of an hour before the last meal, and a second dose of from thirty to fifty at bed-time. In cases in which, without any nervous complaint, there is sleeplessness, owing to some cause of cerebral excitement, as well as in all neuroses, excepting hydrophobia, tetanus, very severe cases of delirium tremens, and some forms of insanity, sleep is almost always induced by that remedy."

Dr. Roberts Bartholow, of Cincinnati, Ohio, after making "experimental investigations into the action of bromide of potassium," publishes the result of his investigations in the Cincinnati *Lancet and Observer*, and the *American Journal of Medical Sciences*, January, 1866, from which we learn the following:

"1st. The bromide of potassium acts by absorption into the blood.

"2d. Its effects are expended upon the nervous centres, or the cerebro-spinal axis.

"3d. Sedation of the heart and circulation, and the various local sedative effects, are secondary results of the impression made upon the nervous centres.

"4th. Its physiological effects are not very decided, and are easily modified by any local disturbance.

"5th. Its therapeutical action is still more decidedly influenced by local morbid processes.

"6th. It is indicated where a sedative to the nervous system is required—in insomnia; in great reflex excitability; nervous and spasmodic affections of the larynx and bronchi; sexual excitement, and in irritable state of the sexual organs.

"7th. It will be effectual in the foregoing conditions, in proportion to the degree in which structural lesions are absent, or, in other words, in proportion to the degree in which these morbid states are functional rather than organic."

Dr. Bartholow also asserts that the bromide possesses none of the peculiar alterant properties of the iodide of potassium.

Dr. Bedford Brown, of Washington, D. C., in an article published in the *Medical Mirror*, and republished in the Richmond *Medical Journal*, in December, 1867, says that "bromine possesses remarkable sedative powers over the nervous system, without many of those serious effects resulting from more powerful narcotics. This gives it additional value in the treatment of the diseases of organs having such varied and external nervous communications as the testes. . . It has a very decided specific effect in the diseases of the entire genito-urinary system." He is of the opinion that this remedy "partakes both of an alterative and sedative character."

The diseases in which bromide of potash has been found beneficial are: hysteria, chorea, epilepsy, epileptiform cases, insomnia, irritable bladder, orchitis, gonorrhœa, croup, whooping-cough, cerebro-spinal meningitis, bronchitis, laryngitis, puerperal convulsions, etc., etc. As these diseases are so different in their character—some being *nervous* and others *inflammatory*—it would appear to the mind of any one that the bromide must possess some other effect than that of an hypnotic or sedative. In its physiological effects when taken into the stomach, especially when given in large doses, it proves irritant to the mucous membrane of the stomach. It is rapidly absorbed into the blood, and may be soon detected in the urine. I think that it acts upon the system by producing contraction of the vessels of the brain and spinal marrow, and of the general nervous system. As this is, I have no doubt, the physiological effect of the medicine, it may be given in *all* diseases in which it is denied to produce that effect.

The doses should be *large.* Not less than twenty, thirty or sixty grains should be given at a time, when it is desired to have its hyposthenic, sedative, hypnotic and calming effect; and its power will be increased by giving a dose of opium a short time before administering the bromide.

When it becomes necessary to use this medicine for some time, it will be necessary to guard against its evil effects, such as eruptions of the skin, debility of the arms and legs, depression of mind, loss of memory, and delusions. I have observed these effects produced in several cases under my care, but they generally subside upon the discontinuance of the medicine.

